# Bilateral and Optimistic Warning Paradigms Improve the Predictive Power of Intraoperative Facial Motor Evoked Potentials during Vestibular Schwannoma Surgery

**DOI:** 10.3390/cancers13246196

**Published:** 2021-12-09

**Authors:** Tobias Greve, Liang Wang, Sophie Katzendobler, Lucas L. Geyer, Christian Schichor, Jörg Christian Tonn, Andrea Szelényi

**Affiliations:** 1 Department of Neurosurgery, University Hospital, Ludwig-Maximilians-University Munich, 81377 Munich, Germany; sophie.katzendobler@med.uni-muenchen.de (S.K.); christian.schichor@med.uni-muenchen.de (C.S.); joerg.christian.tonn@med.uni-muenchen.de (J.C.T.); andrea.szelenyi@med.uni-muenchen.de (A.S.); 2 Department of Neurosurgery, Beijing Tiantan Hospital, Capital Medical University, Nansihuan Xilu 119, Fengtai District, Beijing 100070, China; wangliang@bjtth.org; 3 Institute of Neuroradiology, University Hospital, Ludwig-Maximilians-University Munich, 81377 Munich, Germany; geyer@rzm.de

**Keywords:** cerebellopontine angle, monitoring, intraoperative, evoked potentials, motor, schwannoma, vestibular, facial nerve

## Abstract

**Simple Summary:**

During surgery for vestibular schwannomas, the facial nerve is monitored via motor evoked potentials (facial nerve MEP). The established warning criteria for facial nerve MEP signal changes mostly refer to the ipsilateral side and disregard the contralateral side. Furthermore, the surgeon is warned as soon as the signal of a single facial muscle deteriorates. We examined how the predictive power of the facial nerve MEP would change if we used the percent change in ipsilateral versus contralateral MEP stimulation intensity over time as warning criterion; additionally, if we warned in a novel optimistic manner, a manner in which the surgeon would be warned only if all derived facial muscles deteriorate significantly, as opposed to the traditional method, in which the surgeon is warned as soon as a single muscle deteriorates. We retrospectively compared this approach to actual intraoperative warnings (based on unilateral threshold change, A-trains, and MEP loss) and show that with our method, the facial nerve MEP was significantly more specific and triggered fewer unnecessary warnings.

**Abstract:**

Facial muscle corticobulbar motor evoked potentials (FMcoMEPs) are used to monitor facial nerve integrity during vestibular schwannoma resections to increase maximal safe tumor resection. Established warning criteria, based on ipsilateral amplitude reduction, have the limitation that the rate of false positive alarms is high, in part because FMcoMEP changes occur on both sides, e.g., due to brain shift or pneumocephalus. We retrospectively compared the predictive value of ipsilateral-only warning criteria and actual intraoperative warnings with a novel candidate warning criterion, based on “ipsilateral versus contralateral difference in relative stimulation threshold increase, from baseline to end of resection” (BilatMT ≥ 20%), combined with an optimistic approach in which a warning would be triggered only if all facial muscles on the affected side deteriorated. We included 60 patients who underwent resection of vestibular schwannoma. The outcome variable was postoperative facial muscle function. Retrospectively applying BilatMT, with the optimistic approach, was found to have a significantly better false positive rate, which was much lower (9% at day 90) than the traditionally used ipsilateral warning criteria (>20%) and was also lower than actual intraoperative warnings. This is the first report combining the threshold method with an optimistic approach in a bilateral multi-facial muscle setup. This method could substantially reduce the rate of false positive alarms in FMcoMEP monitoring.

## 1. Introduction

Preservation of facial nerve integrity is of paramount importance during surgical resection of vestibular schwannomas in the cerebellopontine angle [1]. In recent years, advances in intraoperative neuromonitoring and mapping have led to better preservation of the facial nerve, which improves postoperative facial muscle function while maximizing the extent of resection [2].

Monitoring of facial muscle corticobulbar evoked potentials (FMcoMEPs), elicited by transcranial electrical stimulation, is the routine method for continuous facial nerve monitoring during surgery, and was first described by Dong et al. in 2005 [3,4]. Most groups use similar warning criteria for FMcoMEPs as for motor evoked potentials (MEPs) of the extremities, alerting the surgeon if the FMcoMEP amplitude in any of the derived facial muscles decreases by more than 50% [3,4,5,6,7] or if monomorphic high-frequency EMG patterns, called A-train activity, occur [8,9,10].

Although commonly used [11], the amplitude warning criterion has been questioned because high stimulation intensity is required to achieve maximal amplitudes at baseline, which can lead to disruptive patient motion [12], and because amplitude can exhibit marked variability when stimulation is delivered with supra-threshold paradigms, it is difficult to quantify [13]. Alternative approaches were introduced for supratentorial surgery, using an increase in motor threshold on the affected side as a biomarker [14,15]. More recently, Abboud and colleagues have extended the concept of motor threshold elevation as a warning criterion by systematically including motor threshold on the healthy side in the analysis, based on the principle that bilateral and possibly irrelevant changes to the motor threshold caused by anesthesia, brain shift, or pneumocephalus can be filtered out [16,17].

The threshold level method was subsequently applied to FMcoMEPs and infratentorial procedures. Some studies focused on the absolute increase in threshold on the affected, ipsilateral side [18,19,20], whereas others examined the relative increase in stimulation threshold on the affected versus unaffected side [21], similarly to Abboud and colleagues, for supratentorial procedures. While the threshold level method showed good correlation with postoperative facial muscle function in cerebellopontine angle surgery [20], both the unilateral and bilateral warning paradigms still have relatively high false positive and false negative rates, which poses intraoperative risks [18,19,21].

We hypothesized that FMcoMEP warning criteria based on the increase in ipsilateral versus contralateral stimulation threshold could be improved by a novel optimistic approach in which a warning would be issued only if all ipsilateral facial muscles deteriorated beyond a certain cutoff, in contrast to the traditional approach in which a warning is issued as soon as one ipsilateral facial muscle deteriorates.

The optimistic approach has not been previously studied, and its accuracy was unknown, making a prospective study with the novel approach ethically unacceptable.

Therefore, the aim of the study was to retrospectively compare the diagnostic performance of a novel candidate warning criterion, based on bilateral assessment of stimulation threshold increase and the above-mentioned optimistic approach, with two established warning criteria, as follows: one based on the ipsilateral-only stimulation threshold increase and one based on prospectively collected actual intraoperative warnings to the surgeon (Table 1). The outcome measure was facial muscle function, objectified with the House–Brackmann score [22].

## 2. Materials and Methods

### 2.1. Study Design

In this single-center study, 65 consecutive patients (66 procedures) who underwent resection of a vestibular schwannoma in the cerebellopontine angle between January 2016 and March 2018 were screened for inclusion. Five patients were excluded: one was under 18 years of age, two showed peripheral MEP responses in all facial muscles, and two had no valid baseline MEP responses in any facial muscle. The study was approved by the institutional ethics committee (registration number 18-513) and patients gave written consent.

### 2.2. Clinical Data Collection

The size of vestibular schwannomas was classified according to the Hannover classification [2]. Facial muscle function was assessed both preoperatively and postoperatively on day 1, day 7, and day 90. All resections were performed with the patient in a modified park bench position and via a retrosigmoid approach. Total intravenous anesthesia was applied in all procedures. The extent of resection and postoperative residual tumor volume were objectified by magnetic resonance imaging and operative reports.

### 2.3. Outcome Dichotomization

To perform risk stratification, the outcome measure (facial muscle function) had to be dichotomized. We compared two forms of dichotomization:

The first was to dichotomize between “no deterioration in House–Brackmann score” and “any deterioration in House–Brackmann score”.

The second was to dichotomize between “no/mild deterioration” and “relevant deterioration”. No/mild deterioration was defined as either no deterioration or an increase in House–Brackmann score without exceeding an absolute score of III (complete eye closure preserved). Relevant deterioration was defined as deterioration of the House–Brackmann score with an absolute value of IV or higher (including incomplete eye closure). This second form of dichotomization was introduced because incomplete eye closure carries a high risk of secondary complications [23] and because transitions of the House–Brackmann score from one point to the next have been shown to have high interobserver variability [24]. In contrast, incomplete eye closure represents a clearer clinical parameter for dichotomization than small changes in the House–Brackmann score.

Despite the theoretical advantages explained above, we used ROC curve analysis to determine whether this new form of outcome dichotomization is a valid alternative to evaluating stepwise changes in the House–Brackmann score.

### 2.4. Intraoperative Neuromonitoring

Facial nerve monitoring using FMcoMEPs is part of the standard setup at our institution for resection of vestibular schwannomas. Routine monitoring also includes direct facial nerve stimulation, assessment of spontaneous EMG activity, somatosensory evoked potentials of the median and tibial nerve, MEPs of the extremities, and brainstem auditory evoked potentials. Intraoperative neuromonitoring was conducted with the ISIS system (Inomed Co, Emmendingen, Germany). Analysis of evoked potentials was performed using the NeuroExplorer Software V6, Inomed Co, Emmendingen, Germany.

EMG was recorded through subdermal twisted-pair non-insulated straight needle electrodes (15 mm, Spes Medica, Battipaglia, Italy) inserted bilaterally into the orbicularis oculi, orbicularis oris, and mentalis muscles. FMcoMEPs were elicited by transcranial electrical stimulation with corkscrew-type electrodes (Inomed Co, Emmendingen, Germany). Hemispheric stimulation montages (C3-anode/Cz-cathode; C4-anode/Cz-cathode) were chosen. A constant current stimulator was used to elicit anodal short trains (maximum output 250 mA, 4–5 stimuli with 0.4–0.5 ms duration, train repetition rate 0.5 Hz, interstimulus interval 2 ms to 4 ms). To detect peripheral responses, a single stimulus was delivered 40 ms before the pulse train. Responses were recorded from the ipsilateral orbicularis oculi, orbicularis oris, and mentalis muscles. Impedance was typically less than 5 kΩ. FMcoMEP responses were amplified and filtered (50–2500 Hz). The stimulation threshold of FMcoMEPs was defined as the minimum current intensity that elicits a valid motor response in a given facial muscle with an amplitude ≥2 µV, <20% amplitude fluctuation, consistent wave morphology, and an appropriate response latency—consistent with published data [18,25]. The baseline stimulation threshold was established before tumor resection by gradually increasing stimulus intensity, starting at 50 mA, until at least one of the affected muscles responded. The final stimulation threshold was determined in the same manner but after closure of the dura. During tumor resection, transcranial electrical stimulation was performed at intervals of 3 to 5 min, interleaved with the other monitoring modalities.

To optimize FMcoMEPs for changes in stimulation threshold, the intensity of the stimulation current was increased if the amplitude decreased by more than 50% to keep the amplitude stable.

The surgeon was alerted when an FMcoMEP amplitude decrease occurred that required adjustment of stimulation current intensity in any facial muscle, when FMcoMEP responses were transiently or permanently lost, or when A-train activity occurred in the EMG. Whenever warnings were outspoken, resection was stopped, warm irrigation was applied, and if necessary, local nimodipine was administered. If the potentials recovered or spontaneous EMG activity subsided, resection was continued. If recovery did not occur, resection was continued on another part of the tumor, using EMG monitoring and analysis of compound muscle action potentials in response to direct nerve stimulation.

### 2.5. Binary Classification Testing and Statistics

Because the FMcoMEP data were not normally distributed, the median values and the corresponding interquartile range (IQR) were calculated.

To assess the predictive value of FMcoMEPs in relation to postoperative facial muscle function, evoked potentials were evaluated as a diagnostic test in a 2 × 2 contingency table that allowed binary classification tests.

The hazard limits were chosen in accordance with the literature, dichotomizing between “positive test result” and “negative test result”. The hazard limit for FMcoMEP-based ipsilateral versus contralateral difference in stimulation threshold elevation from dura opening to the end of tumor resection (BilatMT criterion) was set at 20% [16,21]. The hazard limit for the increase in FMcoMEP-based ipsilateral stimulation threshold from dura opening to the end of tumor resection (UnilatMT criterion) was set at 20 mA [18].

Intraoperative events that resulted in a surgeon warning were prospectively recorded and evaluated as a single composite criterion and dichotomized between “warning outspoken” and “no warning outspoken”.

Postoperative facial muscle function was used as the outcome parameter in the 2 × 2 contingency table. Table 1 provides an overview of hazard limit values.

The validity of BilatMT was additionally tested by calculating the Spearman correlation coefficient between the change (from baseline to final) in ipsilateral versus contralateral stimulation threshold increase and absolute House–Brackmann score.

The statistical software used for this study was IBM SPSS version 26 (IBM Corp., Armonk, NY, USA).

## 3. Results

### 3.1. Clinical Characteristics and Outcome

**Figure 1:**
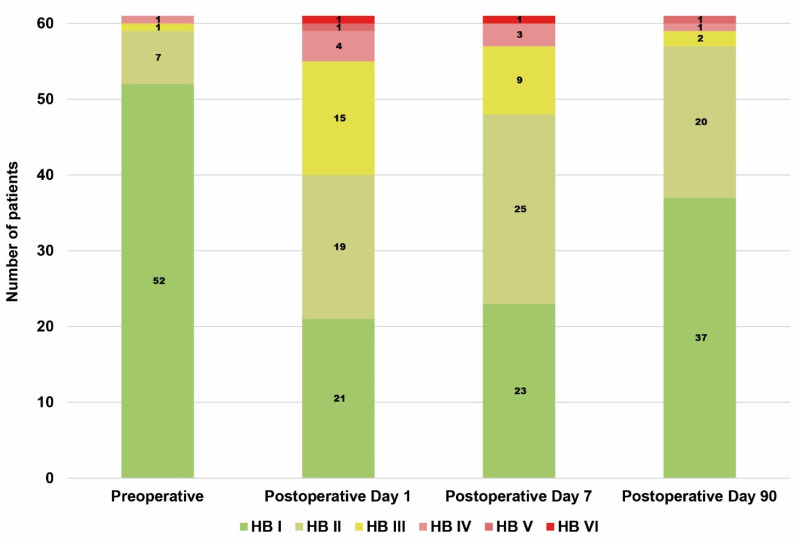
Time plot of facial muscle function. Absolute number of House–Brackmann (HB) scores at each time point are listed inside each bar.

A total of 60 patients (61 procedures) were included in the study. Demographic data are shown in Table 2. Preoperative and postoperative facial muscle function is shown in Figure 1. Preoperatively, facial muscle function was not impaired in 52 (85%) patients. On postoperative day 1, facial muscle function was relevantly impaired (including incomplete eye closure) in 6 (10%) cases. On postoperative day 90, only 2 (3%) patients showed persistent relevant deterioration with incomplete eye closure (Table 3).

### 3.2. Correlation of Postoperative Facial Function to Stimulation Threshold Changes

**Figure 2:**
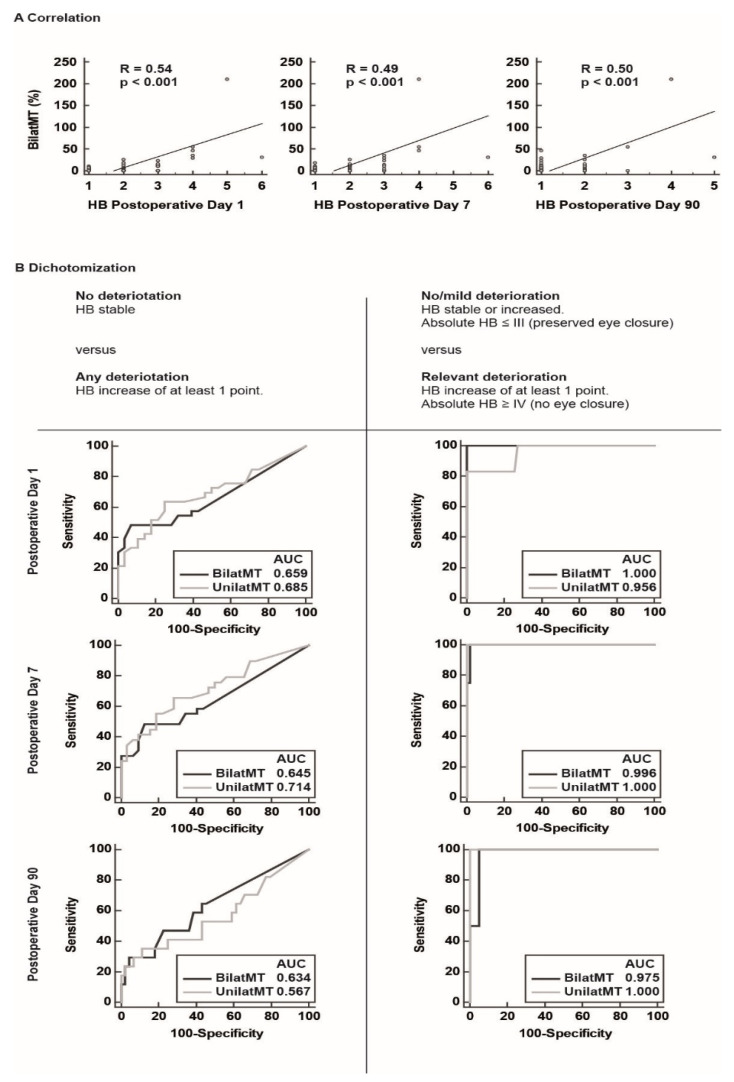
Correlation and ROC curve analysis (**A**) Correlation between change in ipsilateral versus contralateral difference in stimulation threshold increase to elicit FMcoMEPs (BilatMT) and absolute House–Brackmann (HB) score. The correlation was significant for all three time points. (**B**) Comparison of two forms of dichotomization for outcome measurement, for both BilatMT and ipsilateral stimulation threshold increase (UnilatMT). The area under the curve (AUC) was significantly higher for all time points for both BilatMT and UnilatMT.

To test the validity of the new bilateral stimulation threshold criterion BilatMT, the Spearman correlation between the postoperative House–Brackmann score, and the change in stimulation threshold (in %) was calculated. Figure 2A shows that there is a significant correlation between BilatMT and House–Brackmann score for all time points (R = 0.54 at day 1, R = 0.49 at day 6, R = 0.50 at day 90).

### 3.3. Comparison of Outcome Measures for Risk Stratification

For subsequent risk stratification, the postoperative changes in the House–Brackmann score had to be dichotomized. Therefore, we compared which type of dichotomization was more reliably captured by unilateral or bilateral stimulation threshold criteria. The first form of dichotomization was between “no deterioration of the House–Brackmann score” and “any deterioration of the House–Brackmann score”. The second form was dichotomization between “no/mild deterioration” and “relevant deterioration”. Analysis of the ROC curve showed that the area under the curve for the second form of dichotomization between “no/mild deterioration” and “relevant deterioration” was significantly higher for all time points (Figure 2B). Therefore, we chose this form of dichotomization as the outcome measure for risk stratification.

### 3.4. Motor Evoked Potentials and Intraoperative Warnings

Free-running EMG at baseline was available in all patients for the orbicularis oculi, orbicularis oris, and mentalis muscles. A valid response at baseline was obtained in 19 (31%) cases for orbicularis oculi, in 54 (89%) for orbicularis oris, and in 50 (82%) cases for mentalis. Successful baseline FMcoMEP responses were obtained in 16 (26%) patients in all 3 muscles, in 30 (49%) patients in 2 muscles, and in 15 (25%) patients in 1 muscle, with either no response or peripheral responses in the other muscles.

To elicit stable FMcoMEPs on the affected side, the median baseline stimulation threshold was 101 mA (IQR 90–120 mA) for orbicularis oculi, 102 mA (IQR 88–119 mA) for orbicularis oris, and 96 mA (IQR 80–120 mA) for mentalis. The median final stimulation threshold on the affected side was 136 mA (IQR 105–151 mA) for orbicularis oculi, 121 mA (IQR 105–144 mA) for orbicularis oris, and 116 mA (IQR 98–137 mA) for mentalis. Baseline amplitudes were 19 µV (IQR 6–49 µV) for orbicularis oculi, 27 µV (IQR 14–50 µV) for orbicularis oris, and 33 µV (IQR 16–85 µV) for mentalis.

In 29 cases (48% of procedures), intraoperative events resulted in warnings issued to the surgeon. Most warnings (18 of 29) were due to an increase in stimulation intensity that followed a gradual amplitude reduction (as predefined in our stimulation paradigm stated in the methods section), 1 due to permanent loss of FMcoMEPs, 1 due to transient loss of FMcoMEPs, 4 due to A-train activity, and 4 due to transient amplitude fluctuations.

**Figure 3:**
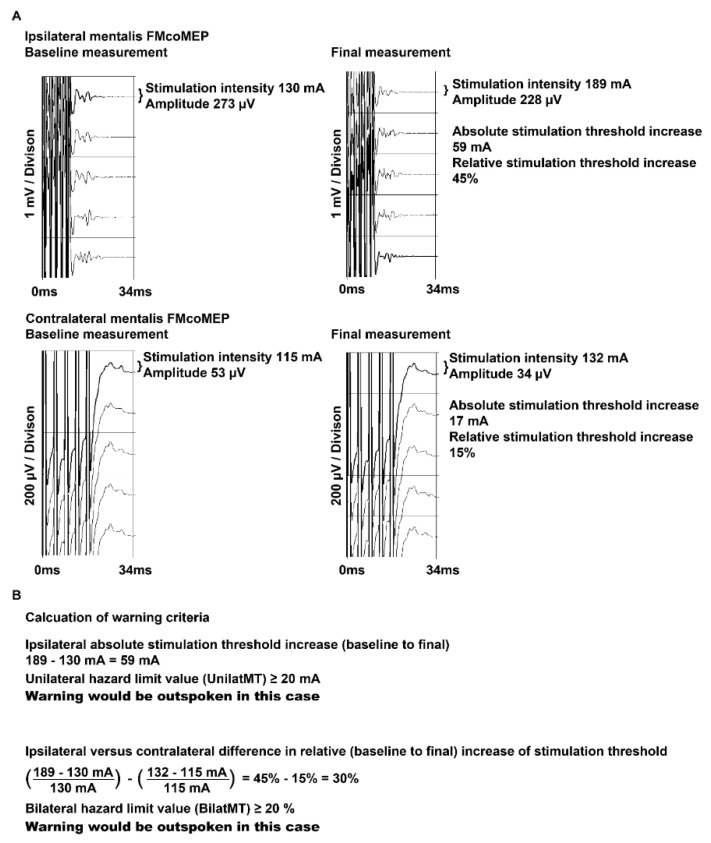
Exemplary IOM data and calculation of warning criteria of patient 11. (**A**) Baseline and final FMcoMEP measurements of the mentalis muscle on the ipsilateral and contralateral sides are shown. For each potential, the amplitude and stimulation intensity are measured. (**B**) Exemplary calculation. The upper calculation refers to the UnilatMT criterion and is calculated by subtracting the baseline from the final stimulation threshold. In this case, the UnilatMT criterion is met because the ipsilateral versus contralateral difference is 59 mA, which is above the 20 mA hazard limit. The bottom calculation refers to the BilatMT criterion and is calculated by subtracting the relative ipsilateral from the relative contralateral increase. In this case, the BilatMT criterion is met because the relative difference is 30%, which is above the 20% hazard limit.

A detailed overview of which patient had intraoperative events leading to warnings and which muscle reached the BilatMT and UnilatMT hazard limits is shown in Table 3. Of note, apart from the differences mentioned in Table 3, there were no major dissimilarities between the first and second surgery in patient 19. For cases 3, 17, and 19-1, there were no noticeable MEP fluctuations during A-train activity. In patient 52, there were MEP fluctuations that began around the same time as A-train activity and resulted in a permanent increase in stimulation threshold. Patient 58 had a preoperative House–Brackmann score of III and patient 59 had a score of IV. Nevertheless, the stimulation threshold at baseline was within the IQR of the cohort (patient 58: orbicularis oris 112 mA, mentalis 118 mA; patient 59: orbicularis oris 99 mA, mentalis 120 mA). Baseline amplitudes in patient 58 were 74 µV (orbicularis oris) and 84 µV (mentalis). In patient 59, they were 18 µV (orbicularis oris) and 59 µV (mentalis). The House–Brackmann scores of both patients gradually improved after surgery as shown in Table 3. Figure 3 shows example ipsilateral and contralateral FMcoMEP recordings.

### 3.5. Risk Stratification

UnilatMT and BilatMT performance measures for the optimistic and traditional approaches were calculated for only 46 (75%) cases in which at least two muscles with valid FMcoMEPs were available at baseline.

For both FMcoMEP-derived criteria, we found a sensitivity of 100%, a negative predictive value of 100%, and a false-negative rate of 0% for all time points and muscles, except in one scenario for UnilatMT (Table 4).

However, substantial differences were found in specificity and false positive rate. For BilatMT, specificity ranged from 76% to 87% for individual muscles and was considerably higher with the optimistic approach (98% at day 1, 95% at day 7, and 91% at day 90) than with the traditional approach (76% at day 1, 74% at day 7, 70% at day 90). If the hazard limit was reached using the optimistic approach (all muscles deteriorated), the postoperative risk of relevant deterioration of facial muscle function was 82% at day 1, 60% at day 7, and 27% at day 90.

For UnilatMT, specificity ranged from 65% to 73% for individual muscles and was higher for the optimistic approach (80% at day 1, 79% at day 7, 75% at day 90) than for the traditional approach (59% at day 1, 57% at day 7, and 55% at day 90). The superior predictive values for BilatMT also translated into an improvement in the false positive rate, especially when the optimistic approach was applied. For BilatMT, it was 2% at day 1, 5% at day 7, and 9% at day 90, whereas for UnilatMT it was 20% at day 1, 21% at day 7, and 25% at day 90 (Table 4).

Intraoperative events leading to a surgeon warning (including transient changes) showed high sensitivity for all time points. Specificity was suboptimal (56%), and the false positive rate was substantially inferior (44%) than for the other criteria (Table 4).

## 4. Discussion

We demonstrated that a novel warning criterion, based on a previously studied bilateral motor threshold criterion (BilatMT ≥ 20%) [16,17,21], in combination with an optimistic approach—in which a warning would be issued only if all facial muscles on the affected side deteriorated—is superior in terms of specificity, false positive rate, and false negative rate, compared with actual intraoperative warnings (including A-trains) and is also superior to the traditionally used method of issuing a warning as soon as one muscle deteriorates beyond the hazard limit.

It also outperformed the FMcoMEP-based ipsilateral-only threshold criterion, particularly in terms of false positive rate. Our data show that bilateral recording and analysis of FMcoMEPs, combined with an optimistic approach, avoids unnecessary warnings to the neurosurgeon without increasing the rate of false negatives.

It remains puzzling why the optimistic approach is superior in predicting facial muscle deteriorations, since one would expect that, for example, A-trains or isolated MEP deteriorations in the orbicularis oculi muscle would have the highest predictive power for eye lid closure. One explanation could be the lack of somatotopy of the facial nerve, as the fibers are not organized into fascicles for specific muscles but are intermingled; a finding previously described in two experimental studies in cats and rats [26,27].

Comparison of our results with the literature shows that the novel criterion outperforms other threshold criteria in corticobulbar MEPs: in a study of 79 patients, a bilateral stimulation threshold criterion was found to outperform unilateral threshold criteria in the early postoperative period, with a sensitivity ranging from 76% to 84% [21]. Two other studies (34 adult patients and 21 children) examining unilateral FMcoMEP stimulation threshold criteria used recordings from multiple muscles but considered only the most “salient” FMcoMEP response for analysis, resulting in selection bias [18,25]. The study with adults showed a sensitivity of 88% and a specificity of 82%, while the study with children showed a sensitivity of 100% and a specificity of 78%. A 2018 study of 95 patients examining a unilateral FMcoMEP stimulation threshold criterion reported a sensitivity of 91% and a specificity of 98%, but only analyzed recordings from the mentalis muscle [28]. As stated in the introduction, the threshold method used for FMcoMEPs was derived from threshold criteria for corticospinal MEPs, where it appears to be a useful addition, increasing specificity by 18% in supratentorial surgery [14,29]. Abboud and colleagues found that using the bilateral threshold criterion in supratentorial surgery further improved sensitivity and specificity to 100% and 97%, respectively [16,17]. Furthermore, a threshold level method might also be superior in spine surgery because the threshold elevation usually precedes amplitude loss by an hour [13].

Amplitude-based warning criteria for FMcoMEPs, although more commonly used than threshold criteria [4,7], were only evaluated in this study as part of the aggregate criterion “intraoperative events leading to a surgeon warning” when the amplitude exhibited a marked fluctuation or dropped to a degree that required us to increase stimulation intensity, as dictated by our inhouse protocol optimized for the threshold method.

Previously, it was shown that compound muscle action potentials of the facial muscle in response to single stimuli represent a technical failure, because bulbar motoneurons require a temporal summation of pulse train stimuli to reach their activation threshold under anesthesia [3,30,31]. Therefore, we used single stimuli, delivered 40 ms before the pulse train, to detect peripheral responses and to rule out direct activation of the facial nerve due to leaking current. Using this method, one must be aware that a short train of transcranial stimuli might alter the excitability of the facial nerve [32], so that the leaking current at sub- or near-threshold intensity might evoke a confounding compound muscle action potential that cannot be distinguished from true FMcoMEPs [33].

This study has several limitations. Although the data were collected prospectively and standardized, the analysis of the novel BilatMT criterion and the optimistic approach was performed retrospectively. Although this retrospective method is common in the exploration of new warning criteria [3,4,6,18,19,21,34,35], it is essential to apply our results to prospective studies. To address this caveat, we also included prospectively collected transient changes that resulted in actual warnings as part of the aggregate warning criterion—“intraoperative events leading to a surgeon warning”. Another limitation is that a reliable FMcoMEP response was obtained from the orbicularis oculi muscle in only 19 cases (31%), either because of a lack of muscle response or because of a peripheral response. The results of the orbicularis oculi muscle (100% sensitivity) must therefore be interpreted with caution. In this context, it is important to note the limitation that successful FMcoMEPs were derived in all 3 muscles in only 16 (26%) patients and in at least 2 muscles in only 30 (49%) patients. Therefore, performance measures using the optimistic and traditional approaches were applied only to this group of 46 (75%) patients. A separate calculation for patients with two or three successfully derived muscles was performed; however, is not presented here, in full, for clarity. In short, the separate calculation shows that BilatMT and the optimistic approach maintains the best predictive power in all of the following 4 separate scenarios: in 16 patients with 3 derived muscles, sensitivity and specificity at 90 days were 100% and 79%, respectively; in 30 patients with 2 derived muscles, sensitivity and specificity at 90 days were 100% and 97%, respectively; in 18 patients with at least 2 derived muscles, including the orbicularis oculi, sensitivity and specificity at 90 days were 100% and 91%, respectively; in 28 patients with at least 2 derived muscles, excluding the orbicularis oculi, sensitivity and specificity at 90 days were 100% and 96%, respectively. This shows that the novel criterion can be successfully applied as soon as two facial muscles are monitored, regardless of whether the orbicularis oculi is successfully obtained or not.

Given the new evidence in the present study, highlighting the relative harmlessness of a single deteriorating muscle, prospective protocols comparing optimistic and traditional approaches are ethically justified and warranted. Improving the false positive rate of this monitoring modality may lead to a safer and more feasible maximal safe resection of vestibular schwannomas in the future.

## 5. Conclusions

We show that, for FMcoMEP warning criteria, a novel optimistic approach (in which a warning would be issued only if all facial muscles on the relevant side deteriorated beyond the hazard limit), combined with a bilateral calculation of hazard limits (relative increase on the ipsilateral side minus relative increase contralaterally), has higher specificity and positive predictive value when retrospectively compared with actual intraoperative warnings (based on ipsilateral-only warning criteria).

This novel approach may help to make resection of vestibular schwannomas and other tumors in the cerebellopontine angle associated with the facial nerve safer, by avoiding false positive and false negative warnings.

With the evidence presented here, prospective studies comparing the novel optimistic with traditional approaches are warranted and ethically justifiable.

## Figures and Tables

**Table 1:** Overview of warning criteria and outcome variables.

Diagnostic Test	Definition	Test Negative	Test Positive
BilatMT optimistic approach	Ipsilateral versus contralateral difference in stimulation threshold increase to elicit FMcoMEPs, from baseline to the end of resection, in all ipsilateral facial muscles.	<20%	≥20%
BilatMT traditional approach	Ipsilateral versus contralateral difference in stimulation threshold increase to elicit FMcoMEPs, from baseline to the end of resection, in one facial muscle.	<20%	≥20%
UnilatMT optimistic approach	Increase in ipsilateral stimulation threshold necessary to elicit FMcoMEPs, from baseline to the end of resection, in all ipsilateral facial muscles.	<20 mA	≥20 mA
UnilatMT traditional approach	Increase in ipsilateral stimulation threshold necessary to elicit FMcoMEPs, from baseline to the end of resection, in one ipsilateral facial muscle.	<20 mA	≥20 mA
Intraoperative warningto the surgeon	Prolonged A-train activity. Amplitude reduction >50% which required increase in stimulation intensity. Transient FMcoMEP loss. Permanent FMcoMEP loss.	No intraoperative warning	Intraoperative warning issued

FMcoMEP—facial muscle corticobulbar motor evoked potential; DNS—direct nerve stimulation.

**Table 2:** Patient characteristics.

Variables	Value
Patients, *n*	60
Procedures, *n*	61
Sex, female	26 (43%)
Age, years	51.5 ± 13.4 (range 21.4–83.5 years)
Tumor volume, cm^3^	19.5 ± 21.4 cm^3^ (range 1.4–113 mL)
Hannover Grading	
T1	0
T2	1 (1.6%)
T3a	11 (18.0%)
T3b	17 (27.9%)
T4a	20 (32.8%)
T4b	12 (19.7%)
Extent of resection	
Gross total resection	48 (78.7%)
Near total resection	12 (19.7%)
Partial resection	1 (1.6%)

Sex, Hannover grading, and extent of resection are noted as count and frequency. Age and tumor volume are noted as mean ± standard deviation.

**Table 3:** Patient data.

				Facial Function (House–Brackmann Score)				Outcome Measure			Ipsilateral Stimulation Threshold Increase to Elicit FMcoMEPs (mA)					Ipsilateral versus Contralateral Difference in Stimulation Threshold Increase (%)					Intraoperative Warnings
Patient Identifier	Tumor volume [mL]	Hanover grading	Extent of resection	Preoperatively	Day 1	Day 7	Day 90	Day 1	Day 7	Day 90	Oculi	Oris	Mentalis	Optimistic	Traditional	Oculi	Oris	Mentalis	Optimistic	Traditional	
1	19	5	NTR	1	2	3	2	0	0	0	9	4	9	4	9	9	4	9	4	9	
2	18	6	GTR	1	2	2	1	0	0	0		12	9	9	12		17	13	13	17	
3	3	4	GTR	1	1	1	1	0	0	0		13	9	9	13		14	9	9	14	AT
4	4	3	GTR	1	3	2	2	0	0	0			2					0			
5	7	5	GTR	1	2	2	1	0	0	0		2	2	2	2		0	0	0	0	
6	30	5	GTR	1	1	1	1	0	0	0			10					8			
7	7	5	GTR	1	3	2	2	0	0	0		12	12	12	12		9	9	9	9	
8	33	5	GTR	1	4	4	1	**1**	**1**	0	**46**	**39**	**48**	**39**	**48**	**55**	**45**	**59**	**45**	**59**	STIM
9	12	4	GTR	1	4	3	2	**1**	0	0	**38**	**47**	**66**	**38**	**66**	**34**	**49**	**91**	**34**	**91**	STIM
10	88	6	PR	1	5	4	4	**1**	**1**	**1**	**170**	**175**	**173**	**170**	**175**	**209**	**230**	**221**	**209**	**230**	LOSS perm
11	8	4	GTR	1	6	6	5	**1**	**1**	**1**	**50**	**60**	**59**	**50**	**60**	**31**	**45**	**30**	**30**	**45**	FLUC
12	31	6	NTR	2	2	2	2	0	0	0		9					7				
13	56	6	GTR	1	3	3	1	0	0	0	17	17	14	14	17	**21**	**21**	10	10	**21**	FLUC
14	2	3	GTR	1	1	1	1	0	0	0		0	**25**	0	**25**		2	**22**	2	**22**	STIM
15	6	4	GTR	1	3	2	2	0	0	0		6	6	6	6		10	10	10	10	
16	4	4	GTR	1	1	1	1	0	0	0		0	0	0	0		7	7	7	7	
17	10	5	GTR	1	1	1	1	0	0	0	2	7	7	2	7	0	0	0	0	0	AT
18	2	3	GTR	1	1	1	1	0	0	0	7	7	7	7	7	0	0	0	0	0	
19-1	19	6	GTR	1	2	2	2	0	0	0	**30**	18	18	18	**30**	13	2	2	2	13	AT
20	4	3	GTR	1	1	1	1	0	0	0		14	0	0	14		0	0	0	0	
21	43	6	GTR	1	3	2	2	0	0	0		11	11	11	11		0	0	0	0	
22	12	4	GTR	1	1	1	1	0	0	0		2	10	2	10		0	8	0	8	
23	113	6	NTR	1	3	2	1	0	0	0		**22**	17	17	**22**		**21**	14	14	**21**	STIM
24	5	3	GTR	1	1	1	1	0	0	0	6	7	6	6	7	7	8	7	7	8	
25	3	3	GTR	1	1	1	1	0	0	0		1	10	1	10		0	0	0	0	
26	4	4	GTR	1	2	2	1	0	0	0		**35**					0				STIM
27	6	4	GTR	1	3	3	1	0	0	0		16	17	16	17		0	0	0	0	
28	35	6	NTR	1	2	2	2	0	0	0	**30**	**30**	**30**	**30**	**30**	4	3	4	3	4	STIM
29	8	2	GTR	1	2	2	1	0	0	0	0	0		0	0	0	0		0	0	
30	30	3	GTR	1	1	1	1	0	0	0		0	0	0	0		0	0	0	0	
31	43	6	NTR	2	3	2	2	0	0	0		18	0	0	18		0	0	0	0	
32	15	5	NTR	1	1	1	1	0	0	0		**40**	**21**	**21**	**40**		**23**	0	0	**23**	STIM
33	26	5	GTR	1	1	1	1	0	0	0			3					0			
34	34	5	GTR	1	1	1	1	0	0	0		3	4	3	4		2	3	2	3	
35	35	5	GTR	2	3	2	2	0	0	0		**20**					14				STIM
36	40	6	NTR	2	2	2	2	0	0	0			9					0			
37	1	3	GTR	2	2	2	1	0	0	0	9	11		9	11	0	0		0	0	
38	43	6	GTR	1	1	1	1	0	0	0	4	**34**	**34**	4	**34**	6	**39**	**39**	6	**39**	STIM
39	2	3	GTR	1	1	1	1	0	0	0	0	**21**	1	0	**21**	0	0	0	0	0	STIM
40	18	5	NTR	1	4	3	1	**1**	0	0		17					**28**				
41	12	5	GTR	1	3	3	1	0	0	0	**40**	**35**	**38**	**35**	**40**	**29**	**23**	**26**	**23**	**29**	STIM
42	19	5	GTR	1	2	2	2	0	0	0		2					**25**				
43	15	5	GTR	1	3	2	2	0	0	0		8	**31**	8	**31**		9	**34**	9	**34**	LOSS trans
44	12	3	GTR	1	2	2	2	0	0	0			0					0			
45	7	4	GTR	1	2	1	1	0	0	0	0					0					
46	17	4	GTR	1	2	2	2	0	0	0		0					0				
47	11	5	GTR	1	2	2	1	0	0	0		**59**	**31**	**31**	**59**		19	0	0	19	STIM
19-2	3	3	GTR	1	3	3	1	0	0	0		**32**	**40**	**32**	**40**		0	12	0	12	STIM
48	2	4	GTR	2	2	2	2	0	0	0		18	**25**	18	**25**		0	0	0	0	STIM
49	13	5	GTR	1	1	1	3	0	0	0		**33**	**43**	**33**	**43**		0	0	0	0	STIM
50	4	4	GTR	2	2	2	2	0	0	0	7	0	0	0	7	0	0	0	0	0	
51	7	4	NTR	1	3	3	1	0	0	0		**21**					0				FLUC
52	19	5	NTR	1	4	4	3	**1**	**1**	0		**53**	**42**	**42**	**53**		**84**	**54**	**54**	**84**	FLUC + AT
53	5	4	GTR	1	1	1	1	0	0	0		9					8				
54	7	4	GTR	1	1	1	2	0	0	0			0					0			
55	78	6	NTR	1	2	1	1	0	0	0		14	14	14	14		17	17	17	17	
56	13	5	NTR	1	1	1	1	0	0	0		**23**	**40**	**23**	**40**		4	**25**	4	**25**	FLUC
57	12	5	GTR	1	2	2	1	0	0	0		**23**	**35**	**23**	**35**		0	**20**	0	**20**	STIM
58	10	4	GTR	3	3	3	2	0	0	0		14	16	14	16		13	14	13	14	LOSS trans
59	34	5	GTR	4	3	2	1	0	0	0		**90**	15	15	**90**		**45**	0	0	**45**	STIM
60	8	4	GTR	1	1	1	1	0	0	0	**27**	19	**30**	19	**30**	0	0	0	0	0	STIM

Cells are marked in bold if any of the diagnostic criteria exceeded the predefined hazard limit or, in case of the outcome measure, if facial function showed relevant deterioration. Outcome measure: dichotomized between 0 = no/mild deterioration (increase/no increase in House–Brackmann score, but absolute value ≤3) and 1 = relevant deterioration (increase in House–Brackmann score, absolute value ≥4); AT—warning due to A-train activity; STIM—warning due to amplitude reduction >50% that obligated us to increase stimulation intensity; LOSS perm—warning due to permanent loss of FMcoMEPs; LOSS trans—warning due to transient loss of FMcoMEPs; FLUC—warning due to amplitude fluctuations.

**Table 4:** Risk stratification.

Criterion	Time Point	Orbicularis Oculi Muscle	Orbicularis Oris Muscle	Mentalis Muscle	Optimistic Approach	Traditional Approach
		Sensitivity, specificity in %				
BilatMT	Day 1	100, 87	100, 85	100, 87	**100, 98**	100, 76
	Day 7	100, 81	100, 82	100, 85	**100, 95**	100, 74
	Day 90	100, 76	100, 79	100, 81	**100, 91**	100, 70
UnilatMT	Day 1	100, 73	83, 69	100, 71	100, 80	100, 59
	Day 7	100, 69	100, 68	100, 70	100, 79	100, 57
	Day 90	100, 65	100, 65	100, 67	100, 75	100, 55
Intraoperative warnings	Day 1	83, 56				
	Day 7	100, 56				
	Day 90	100, 54				
		Positive predictive value, negative predictive value in %				
BilatMT	Day 1	45, 100	43, 100	45, 100	**82, 100**	31, 100
	Day 7	27, 100	28, 100	32, 100	**60, 100**	21, 100
	Day 90	13, 100	14, 100	15, 100	**27, 100**	10, 100
UnilatMT	Day 1	29, 100	23, 97	27, 100	36, 100	21, 100
	Day 7	18, 100	18, 100	19, 100	25, 100	14, 100
	Day 90	9, 100	9, 100	9, 100	12, 100	7, 100
Intraoperative warnings	Day 1	6, 99				
	Day 7	7, 100				
	Day 90	7, 100				
		False negative rate, false positive rate in %				
BilatMT	Day 1	0, 13	0, 15	0, 13	**0, 2**	0, 24
	Day 7	0, 19	0, 18	0, 15	**0, 5**	0, 26
	Day 90	0, 24	0, 21	0, 19	**0, 9**	0, 30
UnilatMT	Day 1	0, 27	17, 31	0, 29	0, 20	0, 41
	Day 7	0, 31	0, 32	0, 30	0, 21	0, 43
	Day 90	0, 35	0, 35	0, 33	0, 25	0, 45
Intraoperative warnings	Day 1	17, 44				
	Day 7	0, 44				
	Day 90	0, 46				

The values in bold indicate the condition with the best results.

## Data Availability

Data available upon reasonable request.
